# Exploring protein structural dissimilarity to facilitate structure classification

**DOI:** 10.1186/1472-6807-9-60

**Published:** 2009-09-19

**Authors:** Pooja Jain, Jonathan D Hirst

**Affiliations:** 1 School of Chemistry, The University of Nottingham, University Park, Nottingham, NG7 2RD, UK

## Abstract

**Background:**

Classification of newly resolved protein structures is important in understanding their architectural, evolutionary and functional relatedness to known protein structures. Among various efforts to improve the database of Structural Classification of Proteins (SCOP), automation has received particular attention. Herein, we predict the deepest SCOP structural level that an unclassified protein shares with classified proteins with an equal number of secondary structure elements (SSEs).

**Results:**

We compute a coefficient of dissimilarity (Ω) between proteins, based on structural and sequence-based descriptors characterising the respective constituent SSEs. For a set of 1,661 pairs of proteins with sequence identity up to 35%, the performance of Ω in predicting shared *Class*, *Fold *and *Super-family *levels is comparable to that of DaliLite *Z *score and shows a greater than four-fold increase in the true positive rate (TPR) for proteins sharing the *Family *level. On a larger set of 600 domains representing 200 families, the performance of *Z *score improves in predicting a shared *Family*, but still only achieves about half of the TPR of Ω. The TPR for structures sharing a *Super-family *is lower than in the first dataset, but Ω performs slightly better than *Z *score. Overall, the sensitivity of Ω in predicting common *Fold *level is higher than that of the DaliLite *Z *score.

**Conclusion:**

Classification to a deeper level in the hierarchy is specific and difficult. So the efficiency of Ω may be attractive to the curators and the end-users of SCOP. We suggest Ω may be a better measure for structure classification than the DaliLite *Z *score, with the caveat that currently we are restricted to comparing structures with equal number of SSEs.

## Background

The increased pace of protein structure determination, due to high-throughput, synchrotron-based X-ray crystallography and multi-dimensional NMR, promises rapid growth in the number of known protein structures [1-3]. Comparison and classification of newly resolved structures contributes to our understanding of the structural architecture, evolution and function of proteins, especially those with low sequence identity to well characterised proteins [4,5]. This information is important for the identification of new protein folds, drug discovery, and phylogenetic analysis of the proteome.

Classification schemes, such as SCOP (Structural Classification Of Proteins) [6] and CATH [7], are well established. SCOP is a curated database and probably the leading classification scheme, providing a comprehensive description of the structural and evolutionary relationships among all protein structures [6]. It classifies about 34,500 structurally resolved proteins into seven major structural levels according to the constituent domains. The highest level being *Class *is followed by *Fold*, *Super-family*, *Family*, *Protein *and *Species *in a hierarchical manner, maintaining one-to-many inter-level relationships. Elements describing the higher levels are based on coarse structural similarity and are limited in number [8]. As we move down the hierarchy, levels become more populated with many specific elements to cater for the increasing evolutionary, structural and functional similarities. The biologically meaningful structural similarities crucial for classification [6] can be detected using constituent secondary structure elements (SSEs), e.g., *α*-helix and *β*-strand, which are the major determinants of protein topology [9,10]. For example, SCOP classification generally depends on the presence of common types of SSEs (at the *Class *level), their topological arrangements and connectivity (at the *Fold *level), structural and functional similarity inferred from a common evolutionary origin (at the *Super-Family *level) and sequential relatedness leading to conserved structural signatures important for protein function (at the *Family *level) [11]. The inclusion of information about SSEs can improve the prediction of protein structural class and fold [12-14].

Specifically, the use of secondary structure content, the proportion of different SSE types and relative arrangement of SSEs has been shown to encode information crucial for predicting protein structural class and fold. Chen et al. [15,16] reported an accuracy of up to 68% for protein fold classification on a set of the 27 most populated folds from SCOP 1.71.

Hierarchical classification of protein structures involves various challenges. The correct assignment of a newly resolved structure to a lower level in the hierarchy is much more difficult than assignment to a higher level [17]. Rearrangement in the classification scheme, especially at the lower levels in the hierarchy, is common [11]. Proteins consisting of multiple structurally independent regions, called domains, pose another challenge. Multiple domains might impart structural and functional variability to the parent protein. Therefore, classification of such proteins may span multiple sub-trees in the hierarchy. A classification scheme that takes into account sequence and structural similarity at the level of constituent domain(s) would be advantageous, especially in the case of convergent evolution, where a domain can be found in evolutionarily unrelated proteins, leading to high structural similarity, despite low sequence similarity [18]. This may help to identify functional analogy, if it exists, among proteins possessing the same domain. Therefore, domains are important to consider for the classification. However, structure classification tends to lag behind structure determination due to the need for personal expertise and expert knowledge leading to the manual and semi-automatic maintenance of the classification schemes.

Recently [11], attention has focused on the automation of SCOP, using established structure and sequence comparison methods, independently or in combination. In general, such efforts towards automation are computationally expensive, algorithmically complex, require optimisation and are prone to errors carried forward from the methods used. Hence, a computationally inexpensive and robust approach that minimises the use of secondary algorithms would be welcome.

State-of-the-art structure comparison and alignment methods, such as Dali [4], have been proposed to classify pairs of proteins to appropriate SCOP structural levels [19]. Dali is used to maintain the Dali database [19], a database of pair-wise comparisons of protein structures deposited in the protein data bank (PDB) [20]. Dali uses the experimentally derived 3D atomic co-ordinates for a pair of proteins to calculate a *Z *score (discussed later) to reflect the extent of similarity between the pair. In this work, the stand-alone version of Dali, DaliLite version 2.4.4 is used.

Our work aims to assign one of the top four SCOP structural levels to an unclassified (newly determined) protein structure. We determine the dissimilarity of the unclassified structure, in terms of a coefficient of dissimilarity (Ω), to those proteins in SCOP with an equal number of SSEs. As a case study, we consider proteins with three SSEs. For a given pair of proteins, Ω takes into account the difference in structural and sequence-based descriptors characterising the constituent SSEs. The structural descriptors are the separation and relative orientation of every pair of SSEs and their types (*α*-helix or *β*-strand), whereas the sequence-based descriptors include the length and the average solvent accessibility of the constituent SSEs. Additional sequence-based descriptors defining the lengths of the paired proteins and sequence identity assigned by DaliLite were also considered, and improve the preliminary results we reported earlier [21]. We compare the accuracy of Ω in predicting the deepest common structural level for a pair of proteins to that of DaliLite *Z *score using empirically determined, independent thresholds for the two scores. Building on this initial assessment, we then compare Ω and *Z *score on a set of 600 domains containing three to six SSEs, representing 200 families from SCOP version 1.73.

## Results

**Figure 1:**
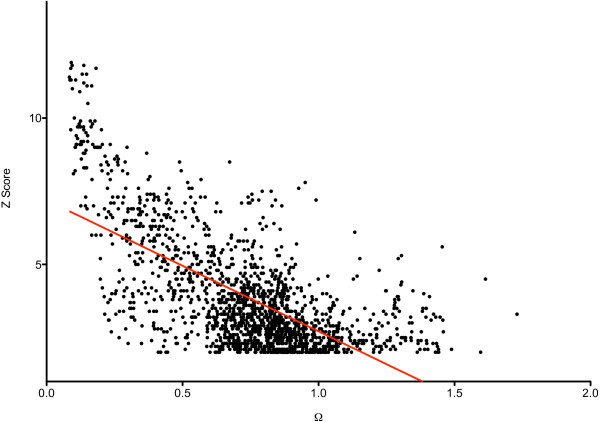
**Correlation of DaliLite *Z *score and coefficient of dissimilarity (Ω) for protein pairs from the DS362 dataset**.

For the 1,661 pairs of proteins, the correlation between *Z *score and Ω was calculated. DaliLite *Z *score measures the extent of similarity, whereas Ω measures the extent of dissimilarity. Therefore, a negative correlation was observed; the squared Pearson's correlation coefficient, *r*^2^, was 0.44 (Figure 1). The correlation between *Z *score and Ω was studied independently for the protein pairs sharing any of the top four structural levels. The highest inverse correlation was observed for the protein pairs sharing a *Family *(*r*^2 ^= 0.42). Surprisingly, for the pairs sharing the *Class*, *Fold *or *Super-family *level the two scores showed negligible correlation (*r*^2 ^= 0.03, 0.01 and 0.08, respectively). In the following we try to rationalise this and evaluate which of the two scores is more appropriate for structural classification.

**Figure 2:**
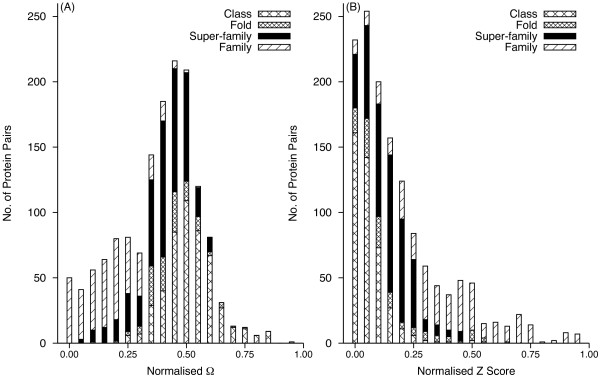
**Distribution of coefficient of dissimilarity (Ω) (A) and DaliLite *Z *Score (B) with respect to different SCOP levels for protein pairs from the DS362 dataset**. Each bin spans an interval of 0.05 of the normalised score.

**Figure 3:**
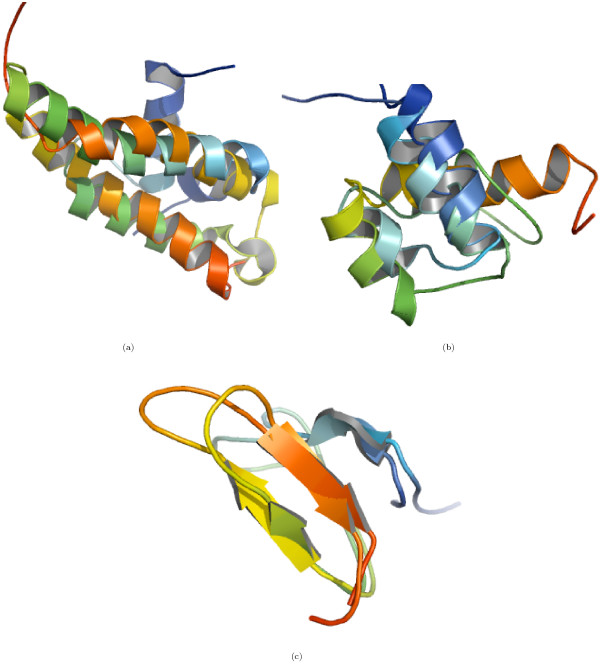
**Proteins from the DS362 dataset with a common (a) Fold **1faf **and **1r73, **sequence identity = 8%, (b) Super-family **1bw5** and **1hcr, **sequence identity = 11% and (c) Family **1cix** and **1kqh, **sequence identity = 21%, for which *Z *score does not identify structural similarity but Ω does**.

The relationship of *Z *score and Ω to the SCOP structural classification was studied through the frequency distribution of protein pairs sharing a structural level (Figure 2). Based on either Ω (Figure 2A) or *Z *(Figure 2B) score, the protein pairs were not distributed discretely between different structural levels. However, protein pairs sharing the *Super-family *and *Family *levels in the hierarchy congregated towards the lower range of Ω, whereas those from the higher level of the hierarchy tended to cluster at the upper range. The reverse was observed in the *Z *score distribution. The number of protein pairs sharing a *Family *increased with Ω, up to a threshold and then decreased. Similarly, the number of protein pairs sharing a *Class *increased slowly at lower values of Ω and then increased sharply when the number of protein pairs sharing a *Family *declined with the further increase in Ω. In contrast, there were some pairs with a common *Family*, which have a very low *Z *score (Table 1). The *Z *score misses structural similarity between some proteins where it is clearly evident by visual inspection and fails to identify for them a common *Fold*, *Super-family *or *Family*. Figure 3 shows some illustrative examples. It appears that *Z *score may not classify correctly when sequence identity is low.

**Table 1:** Selected protein pairs from the DS362 dataset where Ω performs better than *Z *score in predicting the common structural level.

**Shared Level**	**Protein 1**	**Protein 2**	**Ω**	***Z***
**Fold**	1ef4	1iv6	0.57	0.00
	1ef4	1ity	0.52	0.00
	1faf	1r73	0.64	0.02
	1aj3	1rrz	0.64	0.05
	1bby	1hdp	0.57	0.05
	1iv6	1ku3	0.55	0.06
	1ba5	1ku3	0.53	0.08
	1bby	1uhs	0.55	0.09

**Super-family**	1m36	1yuj	0.36	0.00
	1g2h	1ity	0.43	0.00
	1irz	1res	0.33	0.02
	1bf0	1tap	0.44	0.02
	1lfb	1umq	0.40	0.03
	1ig7	1res	0.43	0.03
	1g2h	1iv6	0.43	0.04
	1hcr	1vnd	0.38	0.04
	1bw5	1hcr	0.43	0.06
	1g2h	1jko	0.44	0.07

**Family**	1srk	1yuj	0.14	0.03
	1la4	1oaw	0.25	0.05
	1f43	1hdp	0.08	0.13
	1cix	1oma	0.22	0.02
	1cix	1kqh	0.23	0.07
	1kbe	1tbn	0.22	0.00
	1lqc	1uxc	0.20	0.04
	1hd6	2erl	0.09	0.17

Selected protein pairs where Ω performs better than *Z *score in predicting the common structural level. Normalised scores are listed. *Z *score equal to zero indicates no structural similarity, whereas Ω close to 1 indicates high dissimilarity.

**Figure 4:**
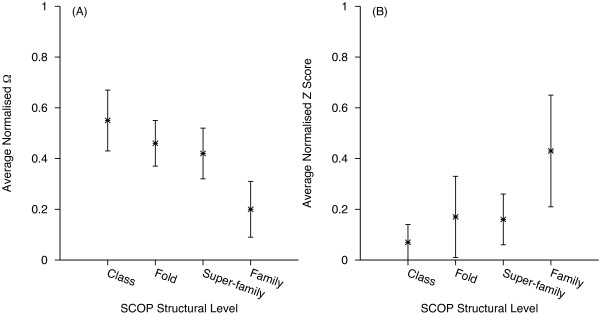
**Mean Ω (A) and *Z *score (B) for the different SCOP levels and standard deviations (error bars) for the DS362 dataset**.

The overlap among the distributions of protein pairs sharing a given structural level was studied (Figure 4). The overlap of *Z *score distributions was greater than those of Ω (Figure 4B). The *Z *score distribution for pairs sharing a *Super-family *completely overlaps the distribution of those sharing a *Fold *and partially overlaps those sharing a *Family*. The *Z *score distribution for pairs sharing a *Class *considerably overlaps the distribution of those sharing a *Fold*. The overlap in the Ω distributions for pairs sharing a given structural level is less extensive, especially among the top three levels. In particular, less overlap was observed for pairs sharing a *Family *(Figure 4A).

These observations suggest that Ω is correlated to the DaliLite *Z *score for the pairs with high sequence identity and is possibly a better measure for the purpose of structure classification. Next, we identify thresholds for Ω that can be used to assign a common structural level for a given pair. To compare the accuracy of Ω for structural classification with that of *Z *score, the two scores were normalised (see Additional file 1, Eqn. S.1; other established statistical and mathematical manipulations that we use are also listed there, for completeness). Hereon, references to Ω and *Z *score are to the normalised scores. The null hypothesis that *no difference exists between the mean normalised value for the two scores for the different structural levels *was tested by the Student's two-sided t-test at a 95% confidence interval, as implemented in the statistical software package R [22]. Very small p-values for the t-test (Table 2) suggest that we can reject the null hypothesis and accept the alternative hypothesis, i.e., there is a significant difference in the mean values of the two scores for pairs sharing a common structural level. Therefore, to predict if a pair shares a given structural level, the mean ± standard deviation (SD) of a normalised score for that level can be suggested as a criterion (Tables 2 and 3). For example, a pair would share a *Class*, if Ω lies within 0.55 ± 0.12 or a *Family*, if Ω lies within 0.20 ± 0.11. Because *Class *and *Family *levels are at the top and at the bottom of the four levels considered, we only require a single threshold for them i.e., Ω ≥ 0.55 and Ω ≤ 0.31, respectively. Considering the overlap between the *Fold *and *Super-family *levels, a single value threshold will not work 100% of the time, but a range may provide some useful predictions. For example, for those pairs where Ω = 0.42 ± 0.10 a common *Super-family *level can be predicted and for pairs where Ω = 0.44 ± 0.09 a common *Fold *level can be predicted. Those pairs for which Ω lies within the overlapping range of super-family and fold thresholds are discussed in the next section. Nevertheless, using these thresholds we have tried to mimic real-world structure classification using the two scores independently in terms of true positive rates (TPR) and false positive rates (FPR) (Table 3). We observe comparable performance of Ω and *Z *score for the top three levels (*Class*, *Fold*, *Super-family*) and a better performance for Ω in classification to the *Family *level. The confusion matrices used to calculate TPR and FPR are provided in Additional file 2. The FPR for classification to the *Fold *level using *Z *score is 38% higher than for Ω. Although the FPR for assigning a common *Family *level using *Z *score is zero, Ω is better, as it has a 75% higher TPR. Correct assignment to the lower levels in the hierarchy is more important than to the higher levels, because assignment to the lower level automatically assigns a pair to all higher levels in the same classification sub-tree.

**Table 2:** Statistical Significance of DaliLite *Z *score and Ω in identifying different SCOP structural levels for the DS362 dataset

**SCOP Level**	**Mean**		**Mean**_***Norm***_		**t-test**	**F-test**	**Max**		**Min**	
	
	***Z***	**Ω**	***Z***	**Ω**	**p-value**_**95%**_	**p-value**_**95%**_	***Z***	**Ω**	***Z***	**Ω**
Class	2.68	0.91	0.07	0.55	10^-16^	10^-1^	7.50	1.60	2.00	0.44
Fold	3.72	0.77	0.17	0.46	10^-16^	10^-8^	8.50	1.22	2.00	0.27
Super-family	3.63	0.72	0.16	0.42	10^-16^	10^-1^	0.23	6.90	1.06	2.00
Family	6.28	0.39	0.43	0.20	10^-7^	0.31	11.90	0.95	2.00	0.08

The statistical significance of difference in the mean of the normalized coefficient of dissimilarity (Ω) and DaliLite *Z *score in terms of Student's two-sided t-test at 95% confidence interval. The small p-values from the t-test indicate a significant difference in the mean value of the two scores for the respective shared SCOP level. The p-values for the F-test reported at 95% confidence interval indicate that the two-sided t-test is valid based on the assumption of equality of the two sample variances. Mean_*Norm *_= normalised mean.

The error in the prediction of a common structural level for a pair of protein was assessed in terms of the variance to mean ratio (VMR) (Additional file 1, Eqn. S.2) and the coefficient of error (CE) (Additional file 1, Eqn. S.3). The former reflects the randomness of a given statistic. VMR close to zero indicates a more even distribution and VMR approaching unity indicates a random distribution. The VMR for DaliLite *Z *score and for Ω (Table 3) shows that the distribution of Ω with respect to different structural classes in SCOP is more uniform than the distribution of *Z *score. This indicates that for another set of proteins, the thresholds suggested for Ω are likely to remain the same, whereas those for *Z *score would vary more. CE is a useful measure to evaluate the precision of the quantitative estimate (thresholds) used for classification. A high value of CE reflects poor precision.

**Table 3:** Descriptive statistics for Ω and *Z *scores for the DS362 dataset

**SCOP Level**	**SD**		**SD**_***Norm***_		**VMR**		**CE**		**TPR**		**FPR**	
	
	***Z***	**Ω**	***Z***	**Ω**	***Z***	**Ω**	***Z***	**Ω**	***Z***	**Ω**	***Z***	**Ω**
Class	0.75	0.17	0.07	0.12	0.21	0.03	0.27	0.19	0.88	0.87	0.31	0.35
Fold	1.62	0.14	0.16	0.09	0.70	0.03	0.43	0.18	0.75	0.69	**0.74**	**0.46**
Super-family	0.93	0.16	0.10	0.10	0.24	0.04	0.26	0.22	0.74	0.70	0.35	0.34
Family	2.21	0.18	0.22	0.11	0.78	0.09	0.35	0.46	0.17	**0.81**	0.00	0.07

Statistics for Ω and *Z *scores for the protein pairs sharing a common SCOP level. The true positive rates (TPR) and false positive rates (FPR) are calculated based on the threshold range derived from the respective normalised *Mean ± SD*. Variance to Mean Ratio (VMR) and Coefficient of Error (CE) statistics give an estimate of how general the thresholds could be and how accurate the suggested thresholds are, respectively. Bold-face type indicates where Ω performs better than *Z *score in assigning pairs to a common *Fold *and *Family*. SD_*Norm *_= normalised standard deviation.

**Figure 5:**
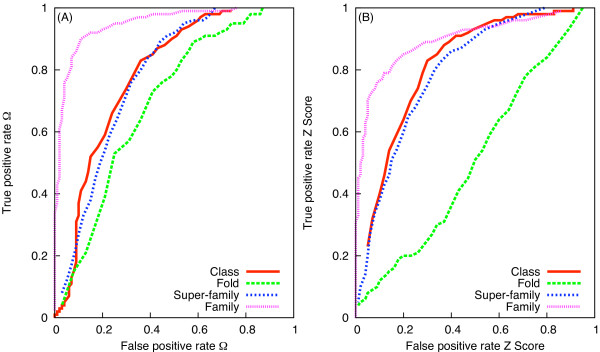
**Sensitivity analysis of TPR and FPR of Ω (A) and *Z *score (B) for the DS362 dataset**.

Whilst the above definition of the classification thresholds seems intuitive, we have also investigated the sensitivity of TPR and FPR to the thresholds, using Receiver Operating Characteristic (ROC) graphs [23]. Figure 5 presents the ROC graphs for Ω and *Z *score. For a given structural level, the TPR and FPR were calculated by increasing the thresholds in intervals of 0.01 from the lowest to the highest normalised scores. For the *Class *and the *Family *level a single threshold was used for the calculation of TPR and FPR. For the *Fold *and the *Super-family *level the interval of 0.01 was used to define maximum and minimum value within the observed maximum and minimum values to calculate the TPR and FPR for the two scores. Because of the overlapping distributions of Ω (and *Z *score) among different structural levels (Figure 4A and 4B), the ROC graphs for protein pairs with a common *Class*, *Fold*, *Super-family *and *Family *levels do not converge to 0 or 1 (Figure 5A and 5B). Nevertheless, it is evident that Ω is more accurate than *Z *score in predicting shared *Family *and *Class *levels. In contrast to *Z *score, Ω can predict the common *Fold *level better than random.

**Figure 6:**
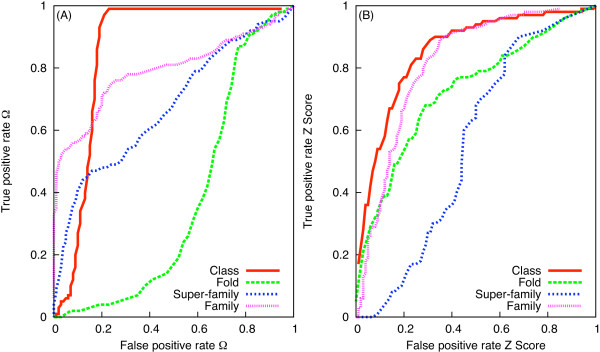
**Sensitivity analysis of TPR and FPR of Ω (A) and *Z *score (B) for the DSF600 dataset**.

The classification to the *Family *level using Ω and *Z *score was investigated on a large dataset of domains (DSF600) representing 200 families (see methods). Pairs of domains consisting of an equal number of SSEs were considered, to determine thresholds for Ω and *Z *score for predicting different structural levels. We have used the range defined by mean ± SD of the normalised scores as the criterion for a given structural level, as reported in Tables 4 and 5. However, such ranges, for the four levels, overlap considerably. The impact of varying single value thresholds is shown by the ROC graphs (Figure 6A and 6B). The statistical significance of classification success of the two scores is reported in Table 4 and the TPR and FPR are shown in Table 5. The first assessment utilising proteins consisting of three SSEs enabled us to identify protocol and relevant measures for fair comparison of Ω and *Z *score. On the DSF600 dataset, the overall trend in the TPR, FPR and statistical significance was similar to the first assessment. Using DSF600 for Ω, the FPR for classification to *Class*, *Fold *and *Super-family *levels decreased relative to the first assessment; the TPR, however, increased for predicting a common *Fold*. For predicting a *Family*, using Ω, the FPR decreased considerably with a slight decrease in the TPR. Using *Z *score, the increase in TPR for classification to the *Family *level occurs at the cost of increase in the FPR. The confusion matrices used to calculate the TPR and FPR are provided in Additional file 3.

**Table 4:** Statistical Significance of DaliLite *Z *score and Ω in identifying different SCOP structural levels for the DSF600 dataset

**SCOP Level**	**Mean**		**Mean**_***Norm***_		**t-test**	**F-test**	**Max**		**Min**	
	
	***Z***	**Ω**	***Z***	**Ω**	**p-value**_**95%**_	**p-value**_**95%**_	***Z***	**Ω**	***Z***	**Ω**
Class	2.96	1.11	0.12	0.64	10^-16^	10^-16^	9.80	1.65	2.00	0.16
Fold	5.01	1.03	0.39	0.30	10^-10^	10^-5^	9.60	1.57	2.00	0.80
Super-family	4.79	1.05	0.24	0.54	10^-11^	10^-2^	13.00	1.33	2.20	0.72
Family	13.10	0.40	0.40	0.30	10^-16^	10^-1^	29.60	1.32	2.00	0.00

The statistical significance of difference in the mean of the normalised coefficient of dissimilarity (Ω) and DaliLite *Z *score in terms of Student's two-sided t-test at 95% confidence interval. The small p-values from the t-test indicate a significant difference in the mean value of the two scores for the respective shared SCOP level. The p-values for the F-test reported at 95% confidence interval indicate that the two-sided t-test is valid based on the assumption of equality of the two sample variances. Mean_*Norm *_= normalised mean.

**Table 5:** Descriptive statistics for Ω and *Z *scores for the DSF600 dataset

**SCOP Level**	**SD**		**SD**_***Norm***_		**VMR**		**CE**		**TPR**		**FPR**	
	
	***Z***	**Ω**	***Z***	**Ω**	***Z***	**Ω**	***Z***	**Ω**	***Z***	**Ω**	***Z***	**Ω**
Class	1.11	0.16	0.14	0.10	0.41	0.02	0.37	0.14	0.89	0.86	0.32	0.18
Fold	1.75	0.11	0.23	0.15	0.61	0.01	0.34	0.11	0.59	0.87	0.17	0.20
Super-family	2.64	0.15	0.24	0.25	1.45	0.02	0.55	0.14	0.28	0.37	0.19	0.16
Family	5.61	0.34	0.20	0.26	2.39	0.29	0.42	0.85	0.45	0.79	0.13	0.39

Statistics for the classification performance of Ω and *Z *scores for the protein pairs sharing a common SCOP level in the DSF600 dataset. Columns have the same meaning as those in Table 3.

**Figure 7:**
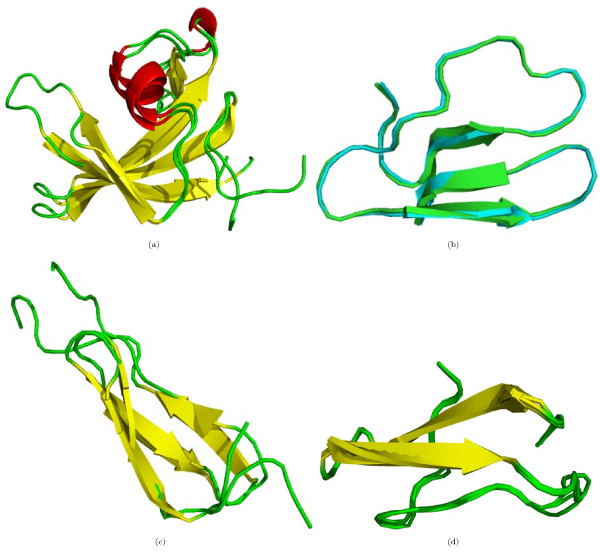
**Domain pairs from the DSF600 dataset with a common family**. DaliLite did not detect any significant structural similarity among the pairs, whereas Ω not only detects the structural similarity but also predicts correctly that the pairs share a family level.(a) d1grja2 and d2eula2, (b) d1cbha_-d2cbha_, (c) d1gl0i_-d1wo9a_ and (d) d1cfwa_, d1dcda_ and d1dfxa_.

DSF600 exhibits significant variation in structural complexity, comprising 200 families, five classes, nine folds and 12 super-families. Nevertheless, Ω seemed robust compared to *Z *score, as indicated by a net increase in the TPR and a net decrease in the FPR in four level classification. Out of 600 possible protein pairs (each of the 200 families is represented by three domains), we can study 505 pairs sharing a *Family*; for the rest, DaliLite cannot detect significant structural similarity. A few of the domain pairs, despite a common *Family*, were not deemed significantly similar by DaliLite (Additional file 4, Table 3), but were correctly predicted by Ω. Figure 7 gives a few illustrative domain pairs. In addition, a single poly-peptide chain can have multiple domains. Such domains have a different classification, if they impart independent functional or structural features, e.g., antibiotic resistant protein domains (d1bl0a1 and d1bl0a2), biotin repressor protein domains (d1biaa1 and d1biaa2) and the hypothetical transporter protein domains (d1v43a1 and d1v43a1). DaliLite cannot handle such domains and fails to detect any structural similarity among them. Clearly, Ω is more suited for the purpose of classification, where it would at least detect a common level from the top of the classification hierarchy.

## Discussion

We have described a computationally inexpensive method for structure classification. For a protein pair with an equal number of SSEs, the method compares structures in terms of structural and sequence-based descriptors characterising the constituent SSEs. The extent of dissimilarity, computed from these descriptors, is used to predict the structural levels from the SCOP hierarchy that the pair shares. Protein pairs sharing a *Class *and *Family *are distinguished by high and low values, respectively, of a dissimilarity coefficient, Ω. However, pairs sharing the intermediate levels, i.e., *Fold *and *Super-family *are not clearly segregated. This can be explained by the Russian doll effect inherent in SCOP and other structure classification schemes [7], where the same sub-structure that constitutes a protein fold exists in the super-family nested within that fold [24].

An inverse correlation was observed between Ω and the similarity score *Z *assigned by DaliLite. We show, however, that Ω is more useful for structure classification than *Z *score. The latter varies more for the protein pairs sharing a SCOP structural level, making it less reliable for structure classification, especially for the *Fold *and *Super-family *levels. A two-sided t-test shows that the mean values for Ω and *Z *score for the protein pairs sharing a common structural level are significantly different. If Ω is used, rather than *Z *score, for assigning a common SCOP level, one can anticipate a six- to eight-fold reduction in the variability of assignment and a 4% to 10% reduction in the chance of making an error (*CE*). It has also been shown that, for a given set of proteins, the threshold range defined around the mean value of Ω more often yields correct structure level assignment compared to the *Z *score (TPR and FPR in Table 3). However, if Ω is used, (instead of *Z *score) 10% more of the predictions for a common *Family *from the existing SCOP families may be incorrect.

The overlapping threshold range of Ω and *Z *score for the *Fold *and *Super-family *levels can be attributed, in part, to the inherent classification caveats that exist in SCOP. Whilst remote homologies exist between the super-families of distinct folds, distant relationships may exist at the *Fold *level. In addition, significant structural variations within the super-family and uncharacterised proteins make it difficult to classify a protein within the current SCOP hierarchy. Often such issues lead to singleton folds, super-families and families. In this context, a recent study discussed improvements to the existing SCOP schema [11] and proposed a redefinition of the *Fold *level and organisation of the *Super-family *level, but did not suggest specific criteria. We have observed that more than 60% of the proteins populating the overlapping threshold range are from the *Homeodomain-like *super-family (SCOP unique identifiers (sunid) = 46689) and the second largest of the folds *DNA/RNA-binding 3-helical bundle *(sunid = 46688). The *Homeodomain-like *super-family has seven singleton families out of 17, whereas the parent fold has nine singleton super-families out of 14. Partners from most of the pairs belong to one of these singleton super-families or folds. Therefore, for classification of pairs in the overlapping regions, redefined thresholds augmented with the sequence identity may be used. A common fold may be predicted for those pairs with sequence identity less than 10% and 0.45 < Ω ≤ 0.55 and for those with sequence identity of 10% or above and 0.31 < Ω ≤ 0.45 a common super-family may be predicted. Those pairs for which redefined thresholds are not applicable, Ω alone may be used for the prediction. It would be possible to check the usefulness of these redefined thresholds upon a major rearrangement of singleton elements in the SCOP hierarchy.

Nevertheless, Ω would still reduce classification space for a given protein and reduce the workload for manual classification.

There are a few outliers (49 pairs) outside the suggested thresholds (Ω = 0.31) for classification to the *Family *level (see Additional file 5, which lists selected PDB codes and names). The classification sub-tree for these pairs reveals that most of them belong to the singleton family of the singleton super-family from the parent fold. For example, the pair 1hry-1l8y, Ω = 0.52, is from the *HMG-Box *family. The pair 1iur-1faf, Ω = 0.57, is from the super-family *Chaperone J-domain*, a singleton super-family containing only family of the same name. This applies to other pairs in which one partner is 1l8y (pairs with 1i11, 1j46, 1j3c, 1hme, 1l8y, 1k99, 1xbl and 1hry) or 1iur or 1faf. Some other outlier pairs include the protein 1tc3 or 1res (pairs with 1hcr, 1jko, 1jj6, 1ijw), from the family *Recombinase DNA-binding domain *that represents mainly the fragments of a whole DNA-binding protein. The structural data for these proteins are in the form of synthetic DNA-protein complexes, which would be different depending on the complexed DNA sequence, structure determination method, and the experimental conditions. Therefore, we presume that for the classification of such fragments considerable expert knowledge would have been taken into account by the SCOP curators. Using Ω, to improve the structure classification of such proteins, information about the structure determination method and nature of the ligand (e.g., DNA, metal ion, etc) should be taken into account. Nevertheless, although these proteins have a DNA-binding domain, they exhibit distinct functions following binding to a specific sequence of DNA, e.g., opening a double helix, relaxing the twist in the DNA during replication, participation in the transposition and recombination. Future rearrangement of the sub-tree of the *Homeodomain-like *super-family is conceivable. Therefore, Ω may potentially help curators in manual assignment. Analysis of the outliers would probably help curators in deciding specific elements to describe the lower levels of the hierarchy.

**Figure 8:**
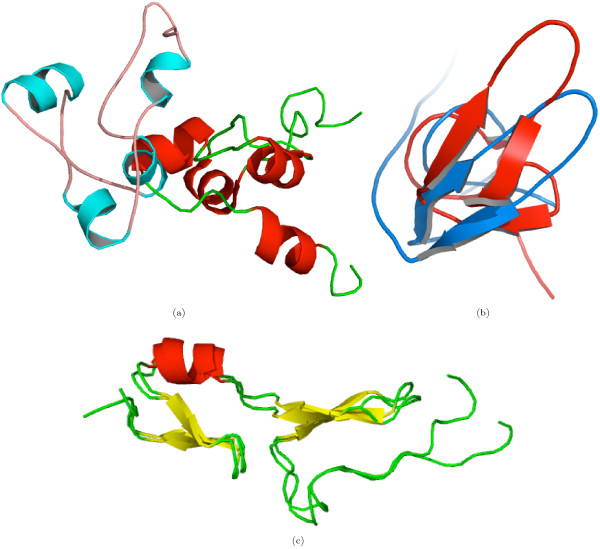
**Pairs of structural variants of the same domain from the DSF600 dataset**. No *Z*-score was assigned to such pairs. (a) d1f4sp_(red) and d2alca_(cyan) (sequence identity = 86%, Ω = 0.30) are the DNA-binding domain of an ethanol regulon transcription factor. The former is DNA bound and the latter is in an unbound state. (b) d1deca_(red) and d1hrti_(blue) (sequence identity = 20%, Ω = 0.21) are the anti-coagulants domains. The structure of the former is derived as an independent domain through solution NMR, while the latter is the part of a complex structure, which was determined through X-ray diffraction. (c) Two variants (d1vgha_ and d2vgha_) of an anti-coagulant heparin binding domain possess high sequence identity.

For classification of the DSF600 dataset, we observed some outliers, arising from the factors discussed above. In addition, however, we also observed a few pairs where one of the domains is either a mutant, or a domain for which structural data is derived independently of the entire protein using a different experimental technique or a domain which was resolve structurally in an unbound state or bound to a ligand. Ω returns very low structural dissimilarity among such variations of the same domain, whereas the *Z *score fails to detect any structural similarity, despite sequence identity above 90%. Figure 8 shows some of the examples of such domain pairs. In comparison to the twilight zone proteins used in the first assessment, the DSF600 dataset contained some homologous proteins. This was advantageous to both Ω and *Z *score, giving a lower FPR. However, at the *Super-family *level there is a decrease in TPR, presumably due to the existence of super-families with high structural similarity [25]. In addition, to the structure of the SCOP hierarchy, the higher homology within the DSF600 dataset might have increased the overlap between the threshold range for Ω and *Z *score to predict the *Fold *and *Super-family *levels. These observations suggest that compared to *Z *score, Ω is robust to the structural diversity as well as to the evolutionary relatedness of the DSF600 domains.

A few possibilities arise from our results. The most important is the identification of various levels in the structural hierarchy based on a set of descriptors without a strict requirement for high sequence identity. This makes the method applicable to twilight zone proteins. Since multiple algorithms and heuristics are not involved, our method does not inherit errors. Presuming that protein folds and families evolve from a common ancestor, our approach may also be useful in studying the emergence of protein fold families [26]. Ω could be used to assess model quality for structure prediction by homology and fold recognition. In addition, Ω facilitates classification of newly determined protein structures, by reducing the classification space to only those structure levels to which the other paired proteins belong. Therefore, the presented methodology can contribute to the existing classification schemes by minimising the need for expert knowledge and manual efforts. A significant correlation is observed between Ω and DaliLite *Z *score. Ω, which is not based on any expert knowledge and is purely dependent on the quality of the descriptors used, is found to be more useful than DaliLite for structure classification. As structural similarity may imply functional similarity, our methodology may find utility in structural class recognition and function prediction [6,27,28].

In its current state, the proposed approach works with structures consisting of an equal number of SSEs. However, on the DSF600 dataset, we have shown its applicability to larger domains and with multi-domain proteins. The SCOP classification is based on the classification of domains and multiple classification sub-trees are possible for a multi-domain protein. Ω can potentially identify such sub-trees. Nevertheless, our approach could be extended to include a combinatorial algorithm guided by the structural descriptors to identify a common core for two domains consisting of a different number of SSEs. It would then be possible to apply our approach to classify large proteins for which the domain boundaries are still unknown. Also, this would help to group proteins with a common core at the *Super-family *or *Fold *level, thereby minimising the thinning of classification hierarchy [29].

## Conclusion

The automation of hierarchical structure classification is important, due to the increasing number of structurally resolved, but unclassified, protein structures. Hierarchical structural classification is prone to inconsistencies, even within the same classification scheme, due to the difference in the amount of information available to define various levels and the limited availability of expert knowledge. Also, over time, the classification changes to accommodate new information, either at the level of structure, evolution or function, e.g., classification of a multi-domain protein under different classification sub-trees, and identification of new relationships with different proteins. In order to cope with the growth in the structural data, automated classification of newly resolved structures to the SCOP hierarchy has been recognised as important. Yet the implemented automated protocol currently within SCOP works at the sequence level and the final, structure-based classification still relies on expert human knowledge, possibly because structural comparison is not as reliable as methods for sequence comparison. In addition, structure comparison is, in general, computationally expensive, involves heuristics and possibly accumulates errors inherited from various secondary algorithms. Hence, a simple approach is needed that can complement the sequence-only automation of classification within SCOP. In this work, we have reported a step towards such an approach.

## Methods

### The Datasets

A set of 362 proteins (DS362, Additional file 6) comprising three SSEs, either helices or strands or both, was derived from the SCOP database [20] version 1.69. The majority of these proteins were single domain proteins. In the case of multi-chain proteins, the first chain was used. The domain boundaries used within a chain were those defined by SCOP. Proteins with invalid DSSP (Definition of Secondary Structure of Proteins) secondary structure assignments [30] or for which DaliLite could not generate a distance matrix were removed. Proteins for which DaliLite did not detect similarity (*Z *score *<*2.0) with any other protein in the dataset were also removed. To avoid bias arising from using sequence similar protein pairs, those detected by DaliLite to have sequence identity above 35% were removed. This generated 1,661 pairs of proteins (205 unique proteins), spanning to four Classes (496 pairs), seven Folds (133 pairs), ten Super-families (472 pairs) and 23 Families (368 pairs) from SCOP. A pair is assigned to one of these four structural levels based on the deepest common structural level between them. For example, proteins in each of the 472 pairs share classification up to the super-family level, but have been assigned to different families.

There were 192 pairs in the dataset with no common level. Analysis of these pairs revealed 67 unique proteins, of which five proteins (1bha, 1m8l, 1m5i, 1lq7 and 2a3d) were classed in SCOP under the *Not a true Class *structural level and one protein (1ijp) to *Membrane and Cell Surface Proteins and Peptide*, a loosely defined SCOP class. These six proteins occurred in about 95% of pairs with no common level. Exclusion of all the pairs having one of these six proteins as one of the partner did not affect the distribution of pairs sharing a given structural level (as above). Therefore, the 192 pairs with no common level were not considered further.

An additional dataset of 600 domains (DSF600, Additional file 7) classified in the SCOP database version 1.73 was derived from the complete set of SCOP domain mappings available from the ASTRAL compendium [31]. We did not use the ASTRAL SCOP domain mappings up to 40% sequence identity, because not enough families were left with at least three domains from different proteins. Using ASTRAL mappings up to 95% sequence identity did not affect the classification results. No intermediate sequence identities were tried. However, to avoid redundant domains, we considered only a single chain of a homomeric protein. To evaluate the proposed methodology on larger domains consisting of more than three SSEs, we include domains comprising up to six SSEs. Three domains were chosen randomly from each of the 50 arbitrarily selected families represented exclusively of domains comprising three, four, five or six SSEs. In this manner, the dataset represents 200 families, enabling us to focus on classification at the *Family *level. We took care to select domains only from true SCOP structural classes. The selected domains represent both single and multi-domain proteins.

### Structural Descriptors

Structural and sequence-based one-dimensional representations of three-dimensional (3D) protein structure have been used for a wide variety of purposes [27,32-35]. Here we use descriptors, which, instead of characterising a protein as a whole, characterise the backbone geometry of the constituent SSEs. The secondary structure assignments were obtained from DSSP. Descriptors were calculated using the C_*α *_co-ordinates of the residues constituting a SSE. These descriptors were grouped into two categories: pair-wise descriptors and individual descriptors. For any two SSEs *i *and *j *in a protein "*a*", two real-valued pair-wise descriptors were the separation,  between the centres of mass (Additional file 1, Eqn. S.4), and the relative orientation, , in terms of the angle between the axes passing through the terminal C_*α *_atoms of the two SSEs (Additional file 1, Eqn. S.5), represented as vectors  and .

**Figure 9:**
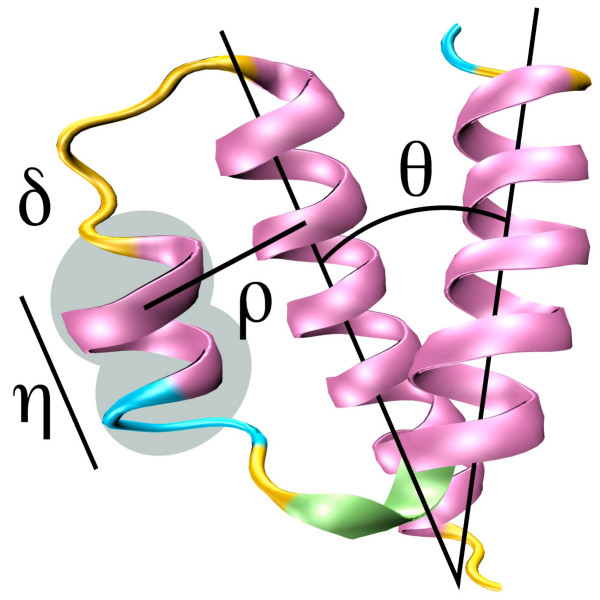
**Pictorial representation of the structural descriptors: separation (*ρ*), orientation (*θ*), solvent accessibility (*δ*) and length (*η*) of the constituent SSEs**.

Three individual descriptors for each SSE are calculated. The average solvent accessibility, *δ*, is the arithmetic mean of the solvent accessibilities assigned by DSSP to each of the residues. The total number of residues constituting a SSE is the length descriptor, *η*, and the SSE type is a binary descriptor, *κ*: *0 *if *α*-helix, 1 otherwise. Figure 9 illustrates these descriptors with reference to a 3D structure. The sequence identity values were assigned by Dali's pair-wise comparison algorithm.

### Coefficient of Dissimilarity

For a pair of proteins *a *and *b*, Ω_*ab *_is defined as the Euclidean distance between the structural and sequence-based descriptors of the two proteins. The root mean square differences (RMSD) with respect to the structural and sequence-based descriptors are derived. For example, for a pair of proteins *a *and *b*, with three SSEs each namely, 1, 2, and 3, the RMSD of every pair-wise SSE distance () is calculated (Additional file 1, Eqn. S.6) and normalised (Additional file 1, Eqn. S.7). Similarly, the normalised RMSD values for other descriptors are used to compute Ω_*ab *_(Eqn. 1). A higher value of Ω indicates greater dissimilarity. Classification to a level in the SCOP hierarchy based on Ω is evaluated.



The coefficient of dissimilarity Ω_*ab *_for two proteins *a *and *b *is defined as the Euclidean distance between *a *and *b *in terms of RMSDs of different structural and sequence-based descriptors calculated for the constituent pairs of SSEs.

### *Z *Score

The Dali *Z *score is an optimised similarity score defined as the sum of equivalent residue-wise C_*α*_-C_*α *_distances among two proteins [4]. Consecutive equivalent residues define so called equivalent structural patterns, generally overlapping hexa-peptides. For the two proteins the largest value of *Z *score corresponds to the optimal set of residue equivalence obtained by permuting the equivalent structural patterns by Monte Carlo optimisation. A higher value of *Z *score indicates greater similarity. A *Z *score below 2.0 is considered as spurious similarity and can be dis-regarded.

## Authors' contributions

JDH proposed the structural descriptors and PJ conceived their use for structural classification. PJ drafted the manuscript and JDH provided advice on presenting results and assisted in drafting the manuscript. Both the authors have read and approved the final version of the manuscript.

## Supplementary Material

Additional file 1 **Equations and statistics**. This file contains the equations and the definitions of statistics used in the study. Click here for file

Additional file 2 **Confusion metrices - the DS362 dataset**. This file contains the confusion matrices used to calculate the True Positive Rate (TPR) and False Positive Rate (FPR) reported for the DS362 dataset. Click here for file

Additional file 3 **Confusion metrices - the DSF600 dataset**. This file contains the confusion matrices used to calculate the True Positive Rate (TPR) and False Positive Rate (FPR) reported for the DSF600 dataset. Click here for file

Additional file 4 **Classification performance of Ω and *Z *score on proteins comprising three to six SSEs**. The pairs of domains from DSF600 data were analysed separately based on the number of comprising SSEs. This file contains the descriptive statistics, statistical significance and ROC graphs for Ω and *Z *score when used to classify domain pairs comprising three to six SSEs, to various structural levels. Selected domain pairs for which the structure similarity was detected by Ω but not by *Z *score are also listed. Click here for file

Additional file 5 **Selected outliers of the pairs sharing a *Family***. This file lists selected outlier protein pairs from the DS362 dataset belonging to a common SCOP *Family*. Click here for file

Additional file 6 **List of domains in the DS362 dataset**. This file lists PDB identifiers for single and multi-domain proteins from the DS362 dataset. In the case of multi-domain proteins the PDB identifier refers to the first chain. Click here for file

Additional file 7 **List of domains in the DSF600 dataset**. This file lists the SCOP identifiers for domains consisting of three, four, five and six SSEs from the DSF600 dataset. Click here for file
