# Supplementary material for: Exploring protein structural dissimilarity to facilitate structure classification
Source: BMC Struct Biol. 2009 Sep 19;9:60. doi: 10.1186/1472-6807-9-60 (PMC2754988; doi:10.1186/1472-6807-9-60)
Supplement: Additional file 1 — Equations and statistics. This file contains the equations and the definitions of statistics used in the study. [file 1472-6807-9-60-S1.pdf]

Additional File - 1

Equations and statistics used in the study

For every pair of proteins the Ω and the Z score have been normalised as:

$$Score_{norm} = \frac{Score - Score_{min}}{Score_{max} - Score_{min}} \tag{S.1}$$

where *Score* can be either Ω or Z score. The maximum and the minimum values across all the pairs used are represented with the respective subscripted acronym.

The variance to mean ration (VMR) for the Ω and the Z score is defined for the protein pairs those sharing a given SCOP structural level.

$$VMR = \frac{Variance}{Mean} \tag{S.2}$$

The coefficient of error defined for protein pairs sharing a given SCOP structural level.

$$CE = \frac{Standard\ Deviation}{Mean} \tag{S.3}$$

The separation of secondary structure elements (SSEs) i and j in a protein a is defined as:

$$\rho_{ij}^{a} = \sqrt{(COM_{Xi} - COM_{Xj})^2 + (COM_{Yi} - COM_{Yj})^2 + (COM_{Zi} - COM_{Zj})^2} \tag{S.4}$$

where $COM_{X(YZ)}$ refers to the $X(YZ)$-coordinate of the centre of mass.

The relative orientation of SSEs i and j in a protein a is defined as:

$$\theta_{ij}^{a} = \cos^{-1} \frac{|V_i||V_j|}{\vec{V_i} \cdot \vec{V_j}} \tag{S.5}$$

where $\vec{V}_{i(j)}$ refers to the vector passing thorough the terminal C_α atoms of the SSE $i(j)$.

The root mean square deviation (RMSD) of the separation among the sequential SSE pairs in proteins a and b is defined as:

$$\rho_{ab}^{rmsd} = \frac{\sqrt{\left(\rho_{12}^{a} - \rho_{12}^{b}\right)^2 + \left(\rho_{13}^{a} - \rho_{13}^{b}\right)^2 + \left(\rho_{23}^{a} - \rho_{23}^{b}\right)^2}}{3} \tag{S.6}$$

The normalised separation RMSD for proteins a and b is defined as:

$$\rho_{ab}^{nrmsd} = \frac{\rho_{ab}^{rmsd} - \rho_{min}^{rmsd}}{\rho_{max}^{rmsd} - \rho_{min}^{rmsd}} \tag{S.7}$$
