# Supplementary material for: Exploring protein structural dissimilarity to facilitate structure classification
Source: BMC Struct Biol. 2009 Sep 19;9:60. doi: 10.1186/1472-6807-9-60 (PMC2754988; doi:10.1186/1472-6807-9-60)
Supplement: Additional file 2 — Confusion metrices - the DS362 dataset. This file contains the confusion matrices used to calculate the True Positive Rate (TPR) and False Positive Rate (FPR) reported for the DS362 dataset. [file 1472-6807-9-60-S2.pdf]

Additional File - 2

Confusion Matrices for the DS362 dataset

Coefficient of Dissimilarity (Ω)

**Class**	Actual		
		Correct	Incorrect
Predicted	Correct	434	343
	Incorrect	62	625

**Fold**	Actual		
		Correct	Incorrect
Predicted	Correct	92	608
	Incorrect	41	**728**

**Super-Family**	Actual		
		Correct	Incorrect
Predicted	Correct	329	373
	Incorrect	143	624

**Family**	Actual		
		Correct	Incorrect
Predicted	Correct	**300**	85
	Incorrect	68	1016

DaliLite Z Score

**Class**	Actual		
		Correct	Incorrect
Predicted	Correct	438	305
	Incorrect	58	668

**Fold**	Actual		
		Correct	Incorrect
Predicted	Correct	100	989
	Incorrect	33	347

**Super-Family**	Actual		
		Correct	Incorrect
Predicted	Correct	350	353
	Incorrect	122	644

**Family**	Actual		
		Correct	Incorrect
Predicted	Correct	66	1
	Incorrect	302	1100
