# Supplementary material for: Exploring protein structural dissimilarity to facilitate structure classification
Source: BMC Struct Biol. 2009 Sep 19;9:60. doi: 10.1186/1472-6807-9-60 (PMC2754988; doi:10.1186/1472-6807-9-60)
Supplement: Additional file 3 — Confusion metrices - the DSF600 dataset. This file contains the confusion matrices used to calculate the True Positive Rate (TPR) and False Positive Rate (FPR) reported for the DSF600 dataset. [file 1472-6807-9-60-S3.pdf]

Additional File - 3

Confusion Matrices for the DSF600 dataset

Coefficient of Dissimilarity (Ω)

**Class**	Actual		
		Correct	Incorrect
Predicted	Correct	939	163
	Incorrect	149	710

**Fold**	Actual		
		Correct	Incorrect
Predicted	Correct	224	350
	Incorrect	33	**1354**

**Super-Family**	Actual		
		Correct	Incorrect
Predicted	Correct	42	295
	Incorrect	63	1500

**Family**	Actual		
		Correct	Incorrect
Predicted	Correct	**401**	575
	Incorrect	104	881

DaliLite Z Score

**Class**	Actual		
		Correct	Incorrect
Predicted	Correct	972	277
	Incorrect	116	596

**Fold**	Actual		
		Correct	Incorrect
Predicted	Correct	152	274
	Incorrect	105	1292

**Super-Family**	Actual		
		Correct	Incorrect
Predicted	Correct	31	369
	Incorrect	80	1481

**Family**	Actual		
		Correct	Incorrect
Predicted	Correct	229	190
	Incorrect	276	1266
