# Supplementary material for: Exploring protein structural dissimilarity to facilitate structure classification
Source: BMC Struct Biol. 2009 Sep 19;9:60. doi: 10.1186/1472-6807-9-60 (PMC2754988; doi:10.1186/1472-6807-9-60)
Supplement: Additional file 4 — Classification performance of Ω and Z score on proteins comprising three to six SSEs. The pairs of domains from DSF600 data were analysed separately based on the number of comprising SSEs. This file contains the descriptive statistics, statistical significance and ROC graphs for Ω and Z score when used to classify domain pairs comprising three to six SSEs, to various structural levels. Selected domain pairs for which the structure similarity was detected by Ω but not by Z score are also listed. [file 1472-6807-9-60-S4.pdf]

Additional File - 4

Table 1 - Statistical Significance of DaliLite Z score and Ω in identifying different SCOP structural levels for the DSF600 dataset

SCOP Level	Mean		Mean$_{Norm}$		Max		Min		t-test	F-test
	Z	Ω	Z	Ω	Z	Ω	Z	Ω	p-value$_{95\%}$	p-value$_{95\%}$
**SSE Count = 3**										
Class	3.08	1.05	0.16	0.29	8.7	1.55	2.0	0.84	10^{-16}	10^{-1}
Fold	5.32	0.93	0.60	0.47	7.5	1.08	2.0	0.8	10^{-5}	10^{-1}
Super-family	3.64	0.89	0.31	0.63	6.8	0.99	2.2	0.72	10^{-1}	10^{-1}
Family	9.92	0.36	0.35	0.31	24.8	1.16	2.0	0.00	10^{-9}	10^{-1}
**SSE Count = 4**										
Class	2.75	1.14	0.10	0.76	9.2	1.44	2.0	0.16	10^{-16}	10^{-16}
Fold	5.11	1.02	0.49	0.32	8.4	1.17	2.0	0.95	10^{-1}	10^{-2}
Super-family	3.39	1.09	0.60	0.46	3.9	1.28	2.6	0.93	10^{-1}	10^{-1}
Family	13.4	0.34	0.44	0.29	26.0	1.15	3.4	0.00	10^{-15}	10^{-1}
**SSE Count = 5**										
Class	2.85	1.26	0.11	0.59	9.8	1.63	2.0	0.74	10^{-16}	10^{-14}
Fold	4.48	1.15	0.33	0.29	9.6	1.57	2.0	0.97	10^{-1}	10^{-1}
Super-family	5.47	1.02	0.51	0.26	6.2	1.17	4.7	0.97	10^{-1}	10^{-1}
Family	13.4	0.47	0.46	0.36	26.2	1.28	2.3	0.01	10^{-11}	10^{-1}
**SSE Count = 6**										
Class	3.23	1.23	0.30	0.41	6.1	1.65	2.0	0.94	10^{-4}	10^{-2}
Fold	4.81	1.04	0.52	0.35	6.9	1.21	2.5	0.95	10^{-1}	10^{-1}
Super-family	6.45	1.16	0.38	0.58	13.0	1.33	2.4	0.94	10^{-3}	10^{-1}
Family	15.3	0.41	0.47	0.30	29.6	1.32	2.6	0.01	10^{-16}	10^{-1}

Table 1: The statistical significance of difference in the mean of the normalised coefficient of dissimilarity (Ω) and DaliLite Z score in terms of Student's two-sided t-test at 95% confidence interval. The small p-values from the t-test indicate a significant difference in the mean value of the two scores for the respective shared SCOP level. The p-values for the F-test reported at 95% confidence interval indicate that the two-sided t-test is valid based on the assumption of equality of the two sample variances. Mean$_{Norm}$ = normalised mean.

Table 2 - Descriptive statistics for Ω and Z scores for the DSF600 dataset

**SCOP Level**	**SD** Z	**SD** Ω	**SD$_{Norm}$** Z	**SD$_{Norm}$** Ω	**VMR** Z	**VMR** Ω	**CE** Z	**CE** Ω	**TPR** Z	**TPR** Ω	**FPR** Z	**FPR** Ω
**SSE Count = 3**												
Class	1.26	0.12	0.19	0.17	0.51	0.01	0.40	0.11	0.80	0.39	0.71	0.41
Fold	1.13	0.06	0.20	0.21	0.23	0.00	0.21	0.06	0.70	0.81	0.09	0.39
Super-family	1.16	0.07	0.25	0.27	0.37	0.00	0.31	0.08	0.70	0.64	0.59	0.26
Family	4.41	0.33	0.19	0.28	1.96	0.29	0.44	0.90	0.09	0.78	0.10	0.71
**SSE Count = 4**												
Class	0.83	0.13	0.11	0.10	0.25	0.01	0.30	0.11	0.86	0.85	0.15	0.12
Fold	1.61	0.05	0.25	0.22	0.50	0.00	0.31	0.04	0.67	0.28	0.25	0.15
Super-family	0.39	0.12	0.30	0.35	0.04	0.01	0.11	0.11	0.62	0.12	0.25	0.19
Family	5.05	0.30	0.22	0.26	1.89	0.27	0.37	0.89	0.66	0.63	0.27	0.18
**SSE Count = 5**												
Class	1.01	0.20	0.13	0.23	0.35	0.03	0.35	0.15	0.79	0.97	0.25	0.17
Fold	2.47	0.19	0.32	0.31	1.35	0.03	0.54	0.16	0.54	0.35	0.26	0.05
Super-family	0.42	0.05	0.28	0.25	0.03	0.00	0.07	0.04	0.50	0.12	0.29	0.13
Family	5.85	0.36	0.24	0.29	2.56	0.27	0.43	0.76	0.17	0.76	0.11	0.25
**SSE Count = 6**												
Class	1.15	0.14	0.28	0.20	0.40	0.01	0.35	0.11	0.56	0.69	0.28	0.29
Fold	1.19	0.08	0.27	0.33	0.29	0.00	0.24	0.08	0.75	0.32	0.59	0.34
Super-family	3.69	0.12	0.35	0.30	2.11	0.01	0.57	0.10	0.28	0.45	0.60	0.40
Family	5.54	0.35	0.20	0.27	2.00	0.30	0.36	0.86	0.63	0.74	0.35	0.60

Table 2: Statistics for Ω and Z scores for the protein pairs sharing a common SCOP level. The true positive rates (TPR) and false positive rates (FPR) are calculated based on the threshold range derived from the respective normalised $Mean \pm SD$. Variance to Mean Ratio (VMR) and Coefficient of Error (CE) statistics give estimate of how generalised the thresholds could be and how accurate are the suggested thresholds, respectively. Bold-face type indicates where Ω performs better than Z score in assigning pairs to a common *Fold* and *Family*. SD$_{Norm}$ = normalised standard deviation.

Table 3 - Ω detects high structural similarity undetected by DaliLite

No. of SSEs	Protein 1	Protein 2	Ω
**3 SSEs**	d1gl0i	d1gl1i	0.01
	d1p7pa	d1pfua	0.02
	d1cbha	d2cbha	0.03
	d1fla_	d1p7pa	0.03
	d1p3ja	d1s3ga	0.06
	d1gl0i	d1wo9a	0.07
**4 SSEs**	d1deca	d1hrti	0.21
	d1f4sp	d2alca	0.30
**5 SSEs**	d1cfwa	d1dfxa	0.11
	d1cfwa	d1dcda	0.13
	d1dcda	d1dfxa	0.19
	d1vgha	d2vgha	0.20
	d1kmxa	d2vgha	0.24
**6 SSEs**	d1grja	d2eula	0.12
	d2etna	d2eula	0.27
	d1a67a	d1cyua	0.36

Table 3: Selected protein pairs for which the structural similarity was undetected by DaliLite, whereas Ω has correctly suggested a shared *Family* for them according to very low dissimilarity threshold as in the main text. Normalised scores are listed.

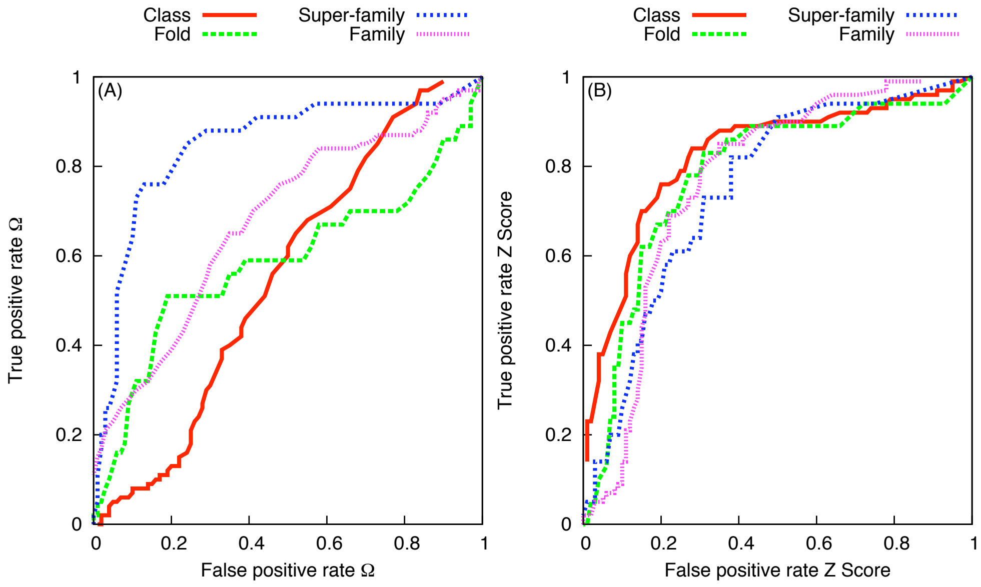

Figure 1: Sensitivity analysis of TPR and FPR for various thresholds of Ω (A) and Z score (B) for domains comprising three SSEs from the DSF600 dataset.

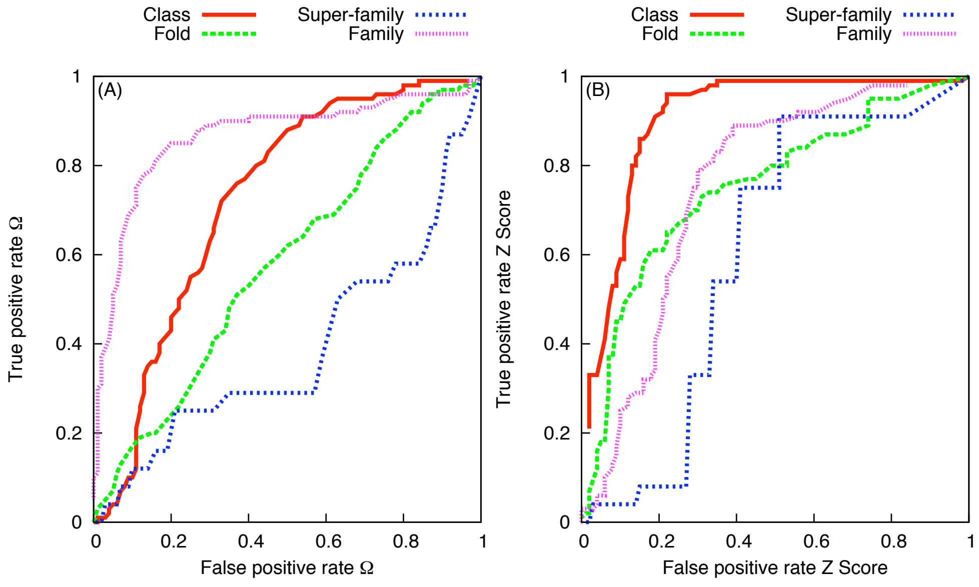

Figure 2: Sensitivity analysis of TPR and FPR for various thresholds of Ω (A) and Z score (B) for domains comprising four SSEs from the DSF600 dataset.

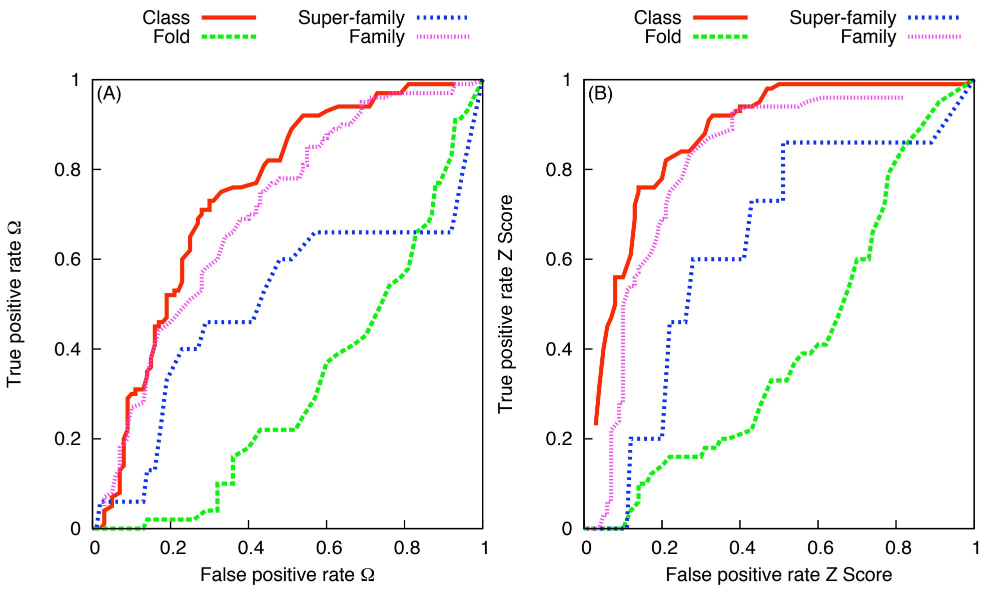

Figure 3: Sensitivity analysis of TPR and FPR for various thresholds of Ω (A) and Z score (B) for domains comprising five SSEs from the DSF600 dataset.

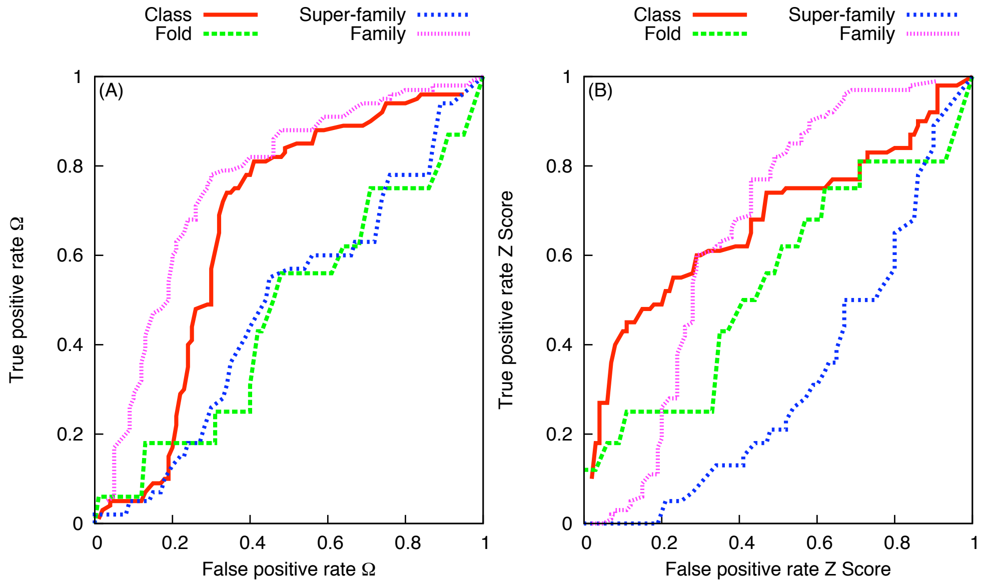

Figure 4: Sensitivity analysis of TPR and FPR for various thresholds of Ω (A) and Z score (B) for domains comprising six SSEs from the DSF600 dataset.
