# Supplementary material for: Exploring protein structural dissimilarity to facilitate structure classification
Source: BMC Struct Biol. 2009 Sep 19;9:60. doi: 10.1186/1472-6807-9-60 (PMC2754988; doi:10.1186/1472-6807-9-60)
Supplement: Additional file 5 — Selected outliers of the pairs sharing a Family. This file lists selected outlier protein pairs from the DS362 dataset belonging to a common SCOP Family. [file 1472-6807-9-60-S5.pdf]

Additional File - 5

Selected outliers of the pairs sharing a *Family*

Prot1	Name	Source	Prot2	Ω_{Norm}	Z Score$_{Norm}$
**1ayg**	Cytochrome C-552	*Hydrogenobacter thermophilus*	1kx7	0.36	0.32
			1c6s	0.43	0.20
			1cyc	0.45	0.21
**1dv0**	DNA repair protein HHR23A C-term UBA Domain	*Homo sapiens*	1pgy	0.36	0.13
			1ify	0.39	0.07
**1ify**	UV excision repair protein Internal UBA Domain of HHR23A	*Homo sapiens*	1pgy	0.35	0.02
			1q02	0.42	0.06
**1hry**	*Homo sapiens* SRY-DNA complex	Synthetic	1hma	0.36	0.27
			1ckt	0.40	0.30
			1aab	0.41	0.22
			1l8y	0.51	0.12
**1l8y**	Upstream binding factor HMG box 5 domain	*Homo sapiens*	1i11	0.35	0.30
			1j46	0.39	0.30
			1j3c	0.43	0.35
			1hme	0.47	0.20
			2lef	0.47	0.29
			1k99	0.48	0.24
**1pgy**	Swa2p UBA Domain	*Saccharomyces cerevisiae*	1f4i	0.39	0.03
			1q02	0.49	0.14
**1q02**	Sequestosome p62 UBA domain	*Homo sapiens*	1f4i	0.44	0.03
			1dv0	0.46	0.01
**1res**	DNA-binding domain of gamma delta resolvase	*Escherichia coli*	1hcr	0.36	0.06
			1jj6	0.37	0.08
			1ijw	0.37	0.09
			1jko	0.41	0.03
			1tc3	0.44	0.18
**1tc3**	DNA-binding domain of Tc3 transposase	*Caenorhabditis elegans*	1hcr	0.40	0.32
			1jko	0.40	0.36
			1jj6	0.41	0.38
			1ijw	0.41	0.38
**1iur**	DnaJ domain of human KIAA0730 protein	*Homo sapiens*	1xbl	0.35	0.11
			1faf	0.57	0.19
**2lfb**	DNA-binding domain of LFB1/HNF1 transcription factor	*Rattus rattus*	1ahd	0.34	0.33
			1pog	0.35	0.16
			1hom	0.35	0.34
			1san	0.37	0.34
			1enh	0.37	0.35
**2lef**	HMG domain	Synthetic	1aab	0.44	0.51
			1ckt	0.44	0.54
