# Supplementary material for: Exploring protein structural dissimilarity to facilitate structure classification
Source: BMC Struct Biol. 2009 Sep 19;9:60. doi: 10.1186/1472-6807-9-60 (PMC2754988; doi:10.1186/1472-6807-9-60)
Supplement: Additional file 6 — List of domains in the DS362 dataset. This file lists PDB identifiers for single and multi-domain proteins from the DS362 dataset. In the case of multi-domain proteins the PDB identifier refers to the first chain. [file 1472-6807-9-60-S6.pdf]

Additional File - 6

List of domains in the DS362 dataset

1a7w
1aab
1acw
1adz
1af8
1ahd
1ail
1aj3
1arf
1aty
1ayg
1azk
1b13
1b2i
1b2j
1b4o
1b6w
1b7i
1ba5
1bby
1bdc
1bds
1be7
1bei
1bf0
1bgk
1bha
1bhi
1bkt
1bl1
1bnb
1boe
1bpi
1bq0
1bq8
1br0
1brf

1bti
1bus
1bw5
1c49
1c56
1c6s
1c6w
1caa
1cbh
1ce3
1cfi
1chl
1chv
1cis
1cix
1ckt
1co4
1cre
1cw6
1cyc
1d5q
1d6b
1dem
1dfe
1dgz
1dkc
1dl6
1dp3
1dtk
1du2
1du6
1du9
1dv0
1dv5
1dwm
1dx7
1dx8
1e0h
1e0l
1e0n
1e4q

1edi
1edx
1ef4
1efe
1egf
1enh
1enk
1erc
1esx
1ews
1eww
1f43
1f4i
1f62
1faf
1fan
1few
1fex
1fh1
1fhh
1fjn
1fme
1ftt
1fu9
1fv5
1g2h
1g6x
1gab
1gat
1ghc
1gjs
1guu
1gv5
1gw4
1gzi
1h02
1h3h
1h5o
1ha8
1hcr
1hd6

1hdp
1hev
1hg7
1hgz
1hly
1hma
1hme
1hn6
1hnr
1hom
1hp8
1hqb
1hry
1hsm
1hta
1hyp
1i11
1i5h
1i6c
1i6z
1i8g
1ic9
1ica
1idy
1iet
1ify
1ig7
1igl
1iio
1ijp
1ijw
1imx
1io6
1irh
1irn
1irz
1ity
1iu5
1iur
1iv6
1iym

1j3c
1j46
1j5n
1j8e
1jab
1jbd
1jc6
1jj6
1jjd
1jjr
1jjs
1jko
1jlz
1jml
1jn7
1ju8
1jv8
1jxc
1k1v
1k36
1k6u
1k81
1k91
1k99
1k9q
1k9r
1kbe
1kbs
1kc4
1kj5
1kma
1knt
1kqh
1kth
1ktx
1ku3
1kun
1kx7
1l3h
1l3o
1l3y

1l4t
1l4v
1l6h
1l8y
1la4
1lcc
1ld5
1lfb
1lg4
1lir
1lq7
1lqc
1lre
1lsi
1lv3
1lwm
1m1q
1m2s
1m36
1m5i
1m62
1m7k
1m8l
1mbe
1mbg
1mbj
1mkn
1mm0
1mmc
1mn3
1msi
1mtx
1mvz
1n1u
1n5g
1n8l
1n8m
1nag
1nbj
1nhm
1njq

1nk2
1nk3
1nre
1o3x
1oa5
1oai
1oaw
1ocp
1og7
1oks
1oma
1oo3
1op1
1ov2
1p8b
1p9z
1pgy
1pir
1pit
1pjv
1pk2
1pnh
1pog
1prb
1pru
1ps2
1q02
1q1v
1q2f
1q2n
1q3j
1q3m
1q8h
1q9b
1qcv
1qkh
1qky
1qlq
1qn0
1qry
1quz

1qxx
1qzp
1r0f
1r4g
1r73
1rb9
1rdg
1res
1rik
1rof
1rrz
1rws
1s24
1san
1scy
1sfv
1shp
1smm
1srk
1ss1
1sxm
1t50
1tap
1tbn
1tc3
1tih
1txa
1txm
1ucs
1ug2
1ugl
1uhs
1ujs
1uk5
1umq
1unc
1ut3
1uua
1uxc
1uxd
1uzc

1v92
1vcx
1vii
1vnd
1w09
1w0b
1whe
1wo9
1xbl
1ypb
1yuj
1zrp
1zwa
2a3d
2ame
2cdx
2cpb
2crd
2erl
2gat
2gf1
2hoa
2lef
2lfb
2pta
2spg
2spz
3bbg
3ci2
3gat
3lri
4gat
4pti
4rxn
5gat
5znf
6gat
7gat
