# Supplementary material for: Exploring protein structural dissimilarity to facilitate structure classification
Source: BMC Struct Biol. 2009 Sep 19;9:60. doi: 10.1186/1472-6807-9-60 (PMC2754988; doi:10.1186/1472-6807-9-60)
Supplement: Additional file 7 — List of domains in the DSF600 dataset. This file lists the SCOP identifiers for domains consisting of three, four, five and six SSEs from the DSF600 dataset. [file 1472-6807-9-60-S7.pdf]

Additional File - 7

List of domains in the DSF600 dataset

3SSE Domains ---- 4SSE Domains ---- 5SSE Domains ---- 6SSE Domains
d1a5ja1.ent ----- d1a7da_.ent ----- d1914a1.ent ----- d14gsa2.ent
d1aala_.ent ----- d1a7ea_.ent ----- d1avza_.ent ----- d14gsb2.ent
d1aapa_.ent ----- d1ab1a_.ent ----- d1avzb_.ent ----- d1a00a_.ent
d1adza_.ent ----- d1acaa_.ent ----- d1b47a2.ent ----- d1a0ba_.ent
d1aila_.ent ----- d1adta1.ent ----- d1b68a_.ent ----- d1a26a1.ent
d1aipc1.ent ----- d1adua1.ent ----- d1bbia_.ent ----- d1a67a_.ent
d1azka_.ent ----- d1adva1.ent ----- d1biaa1.ent ----- d1a8ha1.ent
d1ba5a_.ent ----- d1an1i_.ent ----- d1biaa2.ent ----- d1a90a_.ent
d1bdca_.ent ----- d1apca_.ent ----- d1biba1.ent ----- d1ab7a_.ent
d1bdda_.ent ----- d1au7a2.ent ----- d1bvsa2.ent ----- d1ah5a2.ent
d1br0a_.ent ----- d1auea_.ent ----- d1bz4a_.ent ----- d1aipa2.ent
d1c6wa_.ent ----- d1ayia_.ent ----- d1c2aa1.ent ----- d1aono_.ent
d1c7ya1.ent ----- d1ayja_.ent ----- d1cfwa_.ent ----- d1apja_.ent
d1cbha_.ent ----- d1azya1.ent ----- d1ci4a_.ent ----- d1aqxa2.ent
d1ce3a_.ent ----- d1b22a_.ent ----- d1cooa_.ent ----- d1au1a_.ent
d1cixa_.ent ----- d1b69a_.ent ----- d1cpoa1.ent ----- d1azpa_.ent
d1ck7a6.ent ----- d1b9wa1.ent ----- d1crxa1.ent ----- d1azqa_.ent
d1csb.1.ent ----- d1bb8a_.ent ----- d1cuka2.ent ----- d1b0ba_.ent
d1cuka1.ent ----- d1bhpa_.ent ----- d1d6ri_.ent ----- d1b1ua_.ent
d1d0da_.ent ----- d1bk8a_.ent ----- d1d8ba_.ent ----- d1b23p2.ent
d1deeg_.ent ----- d1bl0a1.ent ----- d1d8la1.ent ----- d1b27e_.ent
d1dfea_.ent ----- d1bl0a2.ent ----- d1d9na_.ent ----- d1b2ue_.ent
d1dgza_.ent ----- d1brwa1.ent ----- d1dcda_.ent ----- d1b59a1.ent
d1du2a_.ent ----- d1brza_.ent ----- d1dfxa2.ent ----- d1b5la_.ent
d1dw9a2.ent ----- d1bxia_.ent ----- d1diva2.ent ----- d1b6aa1.ent
d1dwka2.ent ----- d1c9pb_.ent ----- d1doqa_.ent ----- d1b8aa1.ent
d1e4ya2.ent ----- d1cbna_.ent ----- d1drga1.ent ----- d1b8ca_.ent
d1eaka1.ent ----- d1ceia_.ent ----- d1dvpa2.ent ----- d1b8ka_.ent
d1efub3.ent ----- d1ceja1.ent ----- d1dw9a1.ent ----- d1b8la_.ent
d1ez3a_.ent ----- d1cfma2.ent ----- d1dwka1.ent ----- d1b8za_.ent
d1f4la3.ent ----- d1cgii_.ent ----- d1e6ia_.ent ----- d1b98a_.ent
d1fexa_.ent ----- d1cgji_.ent ----- d1e7db2.ent ----- d1bbba_.ent
d1fjgn_.ent ----- d1ci3m2.ent ----- d1e7la2.ent ----- d1bbxc_.ent
d1fjgt_.ent ----- d1cqtb2.ent ----- d1e8ob_.ent ----- d1bdjb_.ent
d1gata_.ent ----- d1ctma2.ent ----- d1e8oc_.ent ----- d1bhta1.ent
d1gaua_.ent ----- d1cxp.1.ent ----- d1ea8a_.ent ----- d1bipa_.ent

d1gdta1.ent ----- d1d2v.1.ent ----- d1eeja2.ent ----- d1bkba1.ent
d1gl0i_.ent ----- d1d5l.1.ent ----- d1efnb_.ent ----- d1bm9a_.ent
d1gl1i_.ent ----- d1d5ya1.ent ----- d1eg3a2.ent ----- d1bn5a1.ent
d1guua_.ent ----- d1deca_.ent ----- d1eg4a2.ent ----- d1bs2a1.ent
d1hcra_.ent ----- d1dlia1.ent ----- d1en7a2.ent ----- d1bvnt_.ent
d1hf8a1.ent ----- d1dlja1.ent ----- d1f0ya1.ent ----- d1bxea_.ent
d1hfaa1.ent ----- d1dnya_.ent ----- d1f12a1.ent ----- d1c02a_.ent
d1hg2a1.ent ----- d1e0ea_.ent ----- d1f14a1.ent ----- d1c03a_.ent
d1hi7b_.ent ----- d1e3oc2.ent ----- d1f44a1.ent ----- d1c0aa1.ent
d1hjpa1.ent ----- d1e91a_.ent ----- d1f68a_.ent ----- d1c1yb_.ent
d1hnra_.ent ----- d1ee8a1.ent ----- d1faza_.ent ----- d1c3ga1.ent
d1hnwn_.ent ----- d1ejab_.ent ----- d1fbva2.ent ----- d1c3ya_.ent
d1hnwt_.ent ----- d1enia_.ent ----- d1fhua2.ent ----- d1c7ya3.ent
d1hnxn_.ent ----- d1enja_.ent ----- d1fr3a_.ent ----- d1cdqa_.ent
d1hnxt_.ent ----- d1f4sp_.ent ----- d1g0ta2.ent ----- d1cdra_.ent
d1htya1.ent ----- d1f5ep_.ent ----- d1g4da_.ent ----- d1cdsa_.ent
d1huc.1.ent ----- d1ft8e_.ent ----- d1g7da_.ent ----- d1cmia_.ent
d1hwwa1.ent ----- d1g1eb_.ent ----- d1g9la_.ent ----- d1cuka3.ent
d1hxka1.ent ----- d1g2914.ent ----- d1gtda_.ent ----- d1cx8a1.ent
d1i1ga1.ent ----- d1gcca_.ent ----- d1gtdb_.ent ----- d1cyua_.ent
d1i6za_.ent ----- d1gxba1.ent ----- d1guga_.ent ----- d1d8lb2.ent
d1ijwc_.ent ----- d1h59b_.ent ----- d1guna_.ent ----- d1dara3.ent
d1itya_.ent ----- d1hb6a_.ent ----- d1gxie_.ent ----- d1de4c1.ent
d1iv6a_.ent ----- d1hb8a_.ent ----- d1hxda2.ent ----- d1dg1g2.ent
d1iw7f1.ent ----- d1hiai_.ent ----- d1hyia_.ent ----- d1dgsa2.ent
d1j78a3.ent ----- d1hmda_.ent ----- d1hyja_.ent ----- d1e2xa1.ent
d1j7ea3.ent ----- d1hrti_.ent ----- d1i8ta2.ent ----- d1e44a_.ent
d1jeqa1.ent ----- d1i3qf_.ent ----- d1ig4a_.ent ----- d1efya1.ent
d1jj2t_.ent ----- d1i3qj_.ent ----- d1it4a_.ent ----- d1eifa1.ent
d1jjra_.ent ----- d1i50f_.ent ----- d1it5a_.ent ----- d1ekua1.ent
d1jn7a_.ent ----- d1i50j_.ent ----- d1j5ya1.ent ----- d1eqra1.ent
d1k73v_.ent ----- d1i6hf_.ent ----- d1j8ba_.ent ----- d1f3cb_.ent
d1k8av_.ent ----- d1i6hj_.ent ----- d1jb0e_.ent ----- d1f4ka_.ent
d1kdxa_.ent ----- d1i6ve_.ent ----- d1jh4a_.ent ----- d1f7ua1.ent
d1kigi_.ent ----- d1iw7e_.ent ----- d1jida_.ent ----- d1f95a_.ent
d1kqha_.ent ----- d1jvsa1.ent ----- d1jmwa_.ent ----- d1fgpa_.ent
d1ku2a1.ent ----- d1k3wa1.ent ----- d1jzdb2.ent ----- d1fnma3.ent
d1kw2a3.ent ----- d1k3xa1.ent ----- d1k8wa3.ent ----- d1fzva_.ent
d1l0li_.ent ----- d1k5ha1.ent ----- d1kl9a1.ent ----- d1g3pa1.ent
d1l6ja1.ent ----- d1kjka_.ent ----- d1klaa_.ent ----- d1gmna1.ent
d1lr1a_.ent ----- d1kohc2.ent ----- d1kmxa_.ent ----- d1gmoe1.ent

d1lrea_.ent ----- d1kooc2.ent ----- d1kvva_.ent ----- d1grja2.ent
d1lujb_.ent ----- d1l3eb_.ent ----- d1kwia_.ent ----- d1gs0a1.ent
d1lvfa_.ent ----- d1l8ca_.ent ----- d1ky9b2.ent ----- d1gtka2.ent
d1m1eb_.ent ----- d1l9la_.ent ----- d1liha_.ent ----- d1guab_.ent
d1m36a_.ent ----- d1lm3b_.ent ----- d1lnga_.ent ----- d1h9ga1.ent
d1m62a_.ent ----- d1m12a_.ent ----- d1m1ga2.ent ----- d1hcfb_.ent
d1m7ka_.ent ----- d1m7ja2.ent ----- d1m2oa1.ent ----- d1hoea_.ent
d1mn3a_.ent ----- d1mfza1.ent ----- d1m2vb1.ent ----- d1hsta_.ent
d1nh2c_.ent ----- d1moga_.ent ----- d1n5ha_.ent ----- d1huua_.ent
d1nh2d2.ent ----- d1mu5a1.ent ----- d1n5pa_.ent ----- d1hw1a1.ent
d1ni8a_.ent ----- d1mx0a1.ent ----- d1n72a_.ent ----- d1hx5a_.ent
d1nrea_.ent ----- d1n1ia1.ent ----- d1nmra_.ent ----- d1i42a_.ent
d1ns1a_.ent ----- d1nkla_.ent ----- d1nppa2.ent ----- d1i4jb_.ent
d1ntki_.ent ----- d1nsgb_.ent ----- d1npra2.ent ----- d1i94e1.ent
d1nvpc_.ent ----- d1onna1.ent ----- d1nyub_.ent ----- d1ioka3.ent
d1oksa_.ent ----- d1oqya3.ent ----- d1ovna1.ent ----- d1iova1.ent
d1op1a_.ent ----- d1p4qb_.ent ----- d1ovnb1.ent ----- d1iowa1.ent
d1otra_.ent ----- d1pvea_.ent ----- d1pcxa1.ent ----- d1iz6a1.ent
d1p3ja2.ent ----- d1pzna1.ent ----- d1psea_.ent ----- d1j0ra_.ent
d1p7pa3.ent ----- d1qpua_.ent ----- d1pugb_.ent ----- d1jchb_.ent
d1pcpa1.ent ----- d1qzea3.ent ----- d1pugc_.ent ----- d1jj2w_.ent
d1pfua3.ent ----- d1rfya_.ent ----- d1q46a1.ent ----- d1jj2x_.ent
d1pjua1.ent ----- d1rjpa2.ent ----- d1q8ka3.ent ----- d1k73y_.ent
d1pp9i_.ent ----- d1rjqa2.ent ----- d1qasa1.ent ----- d1k73z_.ent
d1ps2a_.ent ----- d1s5qb_.ent ----- d1qcka_.ent ----- d1k8ay_.ent
d1qgwa_.ent ----- d1s78a4.ent ----- d1qpma_.ent ----- d1k8az_.ent
d1qgwb_.ent ----- d1s78b4.ent ----- d1r3fa1.ent ----- d1ksqa_.ent
d1qzpa_.ent ----- d1smye_.ent ----- d1r6wa2.ent ----- d1l0ba2.ent
d1r49a1.ent ----- d1szpa1.ent ----- d1sgva1.ent ----- d1m2vb5.ent
d1r4ga_.ent ----- d1tfkb1.ent ----- d1sotc1.ent ----- d1mc7a_.ent
d1ri7a1.ent ----- d1tfob1.ent ----- d1sska_.ent ----- d1mula_.ent
d1rp3a1.ent ----- d1tn9a_.ent ----- d1t4aa_.ent ----- d1n5ox2.ent
d1s3ga2.ent ----- d1upga_.ent ----- d1tnsa_.ent ----- d1nlta1.ent
d1sb0a_.ent ----- d1us6a_.ent ----- d1ub1a_.ent ----- d1ok0a_.ent
d1se7a_.ent ----- d1v43a1.ent ----- d1usja2.ent ----- d1olza1.ent
d1sp4.1.ent ----- d1v43a2.ent ----- d1vgha_.ent ----- d1oofa_.ent
d1t08b_.ent ----- d1v74b_.ent ----- d1viea_.ent ----- d1ooga_.ent
d1t6oa_.ent ----- d1vmga_.ent ----- d1vifa_.ent ----- d1ovzb2.ent
d1t8ia1.ent ----- d1wjca_.ent ----- d1vlsa_.ent ----- d1ow0c2.ent
d1tapa_.ent ----- d1wjda_.ent ----- d1vq0a2.ent ----- d1oxba_.ent
d1tiha_.ent ----- d1xdoa1.ent ----- d1vzya2.ent ----- d1p3he_.ent

```
d1tl8a1.ent ----- d1xdpa1.ent ----- d1vzyb2.ent ----- d1p8la1.ent
d1ujsa_.ent ----- d1z1ba1.ent ----- d1wama2.ent ----- d1pcxa5.ent
d1unca_.ent ----- d1z1ga1.ent ----- d1wh6a_.ent ----- d1pd1a5.ent
d1wfda_.ent ----- d1z59a1.ent ----- d1wiza_.ent ----- d1pdaa2.ent
d1wgla_.ent ----- d2alca_.ent ----- d1wnla1.ent ----- d1pdgc_.ent
d1wjva1.ent ----- d2aqea1.ent ----- d1wuda1.ent ----- d1pvab_.ent
d1wo9a_.ent ----- d2aqfa1.ent ----- d1x2ia1.ent ----- d1rfaa_.ent
d1wr0a1.ent ----- d2b66h1.ent ----- d1x2la1.ent ----- d1ryja_.ent
d1xb2b1.ent ----- d2b9nh1.ent ----- d1y6ia1.ent ----- d1sjpa3.ent
d1xbta2.ent ----- d2b9ph1.ent ----- d1y8tc1.ent ----- d1t15a2.ent
d1xeqa1.ent ----- d2cc6a1.ent ----- d1yl3k2.ent ----- d1tmqb_.ent
d1xf6a_.ent ----- d2cc7a1.ent ----- d1yvha2.ent ----- d1tola1.ent
d1xx6a2.ent ----- d2cuja1.ent ----- d1z00a1.ent ----- d1tygb_.ent
d1y02a1.ent ----- d2enda_.ent ----- d1z3eb1.ent ----- d1uewa_.ent
d1y0ja1.ent ----- d2etna1.ent ----- d1z3xa1.ent ----- d1uhma_.ent
d1yfba1.ent ----- d2eula1.ent ----- d1z3ya1.ent ----- d1ujua_.ent
d1ysfa1.ent ----- d2f23a1.ent ----- d2a1ja1.ent ----- d1uzja3.ent
d1yxra1.ent ----- d2fapb_.ent ----- d2aw6a1.ent ----- d1v9pa2.ent
d1z0ra1.ent ----- d2g3ka1.ent ----- d2awia1.ent ----- d1vppw_.ent
d1zaea1.ent ----- d2gcca_.ent ----- d2axua1.ent ----- d1we3a3.ent
d2ae9a1.ent ----- d2gdwa1.ent ----- d2bxxb1.ent ----- d1wj4a_.ent
d2aghb1.ent ----- d2gdya1.ent ----- d2bzfa1.ent ----- d1yl3s1.ent
d2bnka1.ent ----- d2gtaa1.ent ----- d2civa1.ent ----- d1yqaa1.ent
d2c5ra1.ent ----- d2gtad1.ent ----- d2ciwa1.ent ----- d1z8la1.ent
d2cbha_.ent ----- d2hfia1.ent ----- d2dgza1.ent ----- d1zud21.ent
d2cfxa1.ent ----- d2huja1.ent ----- d2geca1.ent ----- d2a0ba_.ent
d2df4c1.ent ----- d2im8a1.ent ----- d2gqva1.ent ----- d2b26a1.ent
d2dqnc1.ent ----- d2j9ua1.ent ----- d2hbba1.ent ----- d2b5ub1.ent
d2g5hc1.ent ----- d2j9va1.ent ----- d2iu7a1.ent ----- d2cr5a1.ent
d2hgj81.ent ----- d2o8ra1.ent ----- d2ofja2.ent ----- d2dlna1.ent
d2iu7a2.ent ----- d3gcca_.ent ----- d2p6aa1.ent ----- d2etna2.ent
d2orva2.ent ----- d4hira_.ent ----- d2vgha_.ent ----- d2eula2.ent
```
